# Blood Vessels Pattern Heparan Sulfate Gradients between Their Apical and Basolateral Aspects

**DOI:** 10.1371/journal.pone.0085699

**Published:** 2014-01-22

**Authors:** Liat Stoler-Barak, Christine Moussion, Elias Shezen, Miki Hatzav, Michael Sixt, Ronen Alon

**Affiliations:** 1 Department of Immunology, the Weizmann Institute of Science, Rehovot, Israel; 2 Institute of Science and Technology (IST), Klosterneuburg, Austria; Beth Israel Deaconess Medical Center, Harvard Medical School, United States of America

## Abstract

A hallmark of immune cell trafficking is directional guidance via gradients of soluble or surface bound chemokines. Vascular endothelial cells produce, transport and deposit either their own chemokines or chemokines produced by the underlying stroma. Endothelial heparan sulfate (HS) was suggested to be a critical scaffold for these chemokine pools, but it is unclear how steep chemokine gradients are sustained between the lumenal and ablumenal aspects of blood vessels. Addressing this question by semi-quantitative immunostaining of HS moieties around blood vessels with a pan anti-HS IgM mAb, we found a striking HS enrichment in the basal lamina of resting and inflamed post capillary skin venules, as well as in high endothelial venules (HEVs) of lymph nodes. Staining of skin vessels with a glycocalyx probe further suggested that their lumenal glycocalyx contains much lower HS density than their basolateral extracellular matrix (ECM). This polarized HS pattern was observed also in isolated resting and inflamed microvascular dermal cells. Notably, progressive skin inflammation resulted in massive ECM deposition and in further HS enrichment around skin post capillary venules and their associated pericytes. Inflammation-dependent HS enrichment was not compromised in mice deficient in the main HS degrading enzyme, heparanase. Our results suggest that the blood vasculature patterns steep gradients of HS scaffolds between their lumenal and basolateral endothelial aspects, and that inflammatory processes can further enrich the HS content nearby inflamed vessels. We propose that chemokine gradients between the lumenal and ablumenal sides of vessels could be favored by these sharp HS scaffold gradients.

## Introduction

Chemokines are structurally related chemotactic cytokines which guide the adhesion and motility of many cell types including immune cells [1], [2]. Based on *in vitro* studies, it has been traditionally believed that chemokines guide leukocyte chemotaxis via gradients of soluble molecules [3]. Nevertheless, the most fully established *in vivo* example of chemokine function is leukocyte arrest on blood vessels, a chemokine-driven process that requires immobilized chemokines, but does not depend on a chemokine gradient or on soluble chemokines [4].

Unlike lipid chemoattractants, which operate in their membrane bound lipophilic forms [5]–[7], most chemokines share high affinity binding sites for HS glycosaminoglycans (GAGs) [8]. These sites do not overlap or compete with chemokine binding to their G-protein coupled receptors (GPCRs) [8], [9], and thus, chemokine presentation by HS scaffolds not only retains, but in many cases augments, GPCR signaling on adherent or motile leukocytes [10], [11]. The *in vivo* relevance of HS dependent presentation of chemokines to immune cells recruited by blood vessels has been demonstrated either indirectly by competition assays [12] or directly, by *in vivo* or *ex vivo* enzymatic removal of HS moieties [13]–[15]. Genetic evidence for the key roles of HS GAGs in vascular presentation of key leukocyte chemokines to adherent and migratory leukocytes was provided by two milestone studies [13], [16]. *In vivo* involvement of endothelial HS in inflammation was first demonstrated by endothelial-targeted ablation of the enzyme required for N-sulfation of HS GAGs, which resulted in attenuated neutrophil infiltration to sites of inflammation [16]. In a subsequent study, conditional endothelial specific deletion of the main enzyme that initiates HS GAG biosynthesis, Ext1, led to total loss of HEV HS, eliminated both CCL21 and CCL2 presentation on resting and inflamed lymph nodes, respectively, and reduced the presentation of the neutrophil-specific chemokine, CXCL1, on inflamed skin vessels [13]. Loss of lymphatic endothelial HS in Ext1-deficient lymphatic vessels also impaired both CCL21 presentation and DC migration towards and across lymphatic endothelial barriers [13].

The *in vivo* requirement for HS-dependent presentation of immobilized chemokines to enable directional leukocyte motility in interstitial spaces was recently demonstrated [14]. The CCR7 chemokine, CCL21, was shown to form a steep gradient within the perilymphatic dermal interstitium and to attract dermal dendritic cells towards lymphatic vessels only when immobilized on HS scaffolds and presented in an haptotactic gradient [14]. Interestingly, this haptotactic CCL21 gradient, promoted highly directional DC migration, but neither its formation nor stability required an additional gradient of HS scaffolds [14]. Similarly, steep gradients of immobilized CXCL8 that require HS scaffolds can be generated *in vivo*, even though the HS containing surfaces these chemokine gradients are generated on contain uniform HS densities [17]. In lymph nodes, CCL21 gradients within the border of B follicles and the T cell zone also form independently of HS gradients [18]. These observations collectively suggest that in tissues, gradients of chemokines immobilized on HS can be generated and maintained primarily by confined chemokine production by a single cell or group of adjacent cells.

In spite of these advances in the field of chemokine-directed leukocyte migration, it is still unclear how chemokine gradients form between basolateral and apical aspects of blood vessels to promote directional leukocyte emigration across these vessels. On one hand, the extensive blood flow exerted on apically bound endothelial chemokines may fully account for the formation of a net chemokine gradient between the lumenal (apical) and ablumenal (basolateral) aspects of blood vessels. On the other hand, there could be a second, non-mutually exclusive, mechanism that helps sustain steep chemokine gradients between apical and sublumenal aspects of endothelial barriers. To identify such a mechanism, we employed several immunohistochemical approaches to study the distribution of HS around post capillary venules. We found that different post capillary venules known to support massive leukocyte trafficking pattern steep gradients of HS moieties between their lumen and the basolateral aspects. Notably, these HS gradient can be further amplified during inflammation by the massive secretion of ECM proteoglycans decorated with HS. These are in turn incorporated into the basal lamina, which is remodeled by both the post capillary venules and their surrounding pericytes to provide HS GAG “carpets”, on which both HS-binding inflammatory chemokines and cytokines can be potentially presented to recently extravasating leukocytes.

## Materials and Methods

### Animals

Female C57BL/6 wild-type mice were purchased from Harlan laboratories (Rehovot, Israel). Heparanase knock-out mice were generated and backcrossed as previously described [19], and were a gift from Prof. I. Vlodavsky (Technion, Haifa). ROSA^mT/mG^ double-fluorescent Cre reporter mice [20] and CD11c-Cre transgenic mice [21] were purchased from Jackson laboratory. ROSA^mT/mG^ transgenic mice [20] express membrane-targeted tandem dimer Tomato (mT) prior to Cre-mediated excision, and membrane-targeted green fluorescent protein (mG) after excision. The ROSA^mT/mG^ strain was crossed with the CD11c-Cre transgenic mouse strain [21]. Mice were used at 8–12 weeks of age. All inflammatory *in vivo* procedures were approved by the Animal Research Committee at the Weizmann Institute of Science.

### Reagents and antibodies

Anti-HS monoclonal mouse IgM clone 10E4 was purchased from Seikagaku Corporation (Tokyo, Japan) and from USBiological Life Sciences (Salem, MA), goat anti-mouse VE-cadherin was from R&D Systems (Minneapolis, MN), the BS1-B4 lectin, rabbit anti-mouse laminin and FITC or Cy3 conjugated mouse monoclonal antibodies to α-SMA were from Sigma-Aldrich (St. Louis, MO). The MECA-79 hybridoma was a gift from Jean-Philippe Girard (IPBS, Toulouse, France). Rabbit anti-mouse LYVE1 was from Abcam (Cambridge, UK). The secondary antibody Alexa-647 goat anti-mouse IgM was purchased from Molecular Probes (Life Technologies, Carlsbad, CA). The secondary antibodies donkey anti-mouse IgM Alexa-488 or 647, donkey anti-goat Alexa-488 or 647, and donkey anti-rabbit TRITC were purchased from Jackson Immunoresearch (West Grove, PA). Hoechst 33342 and TRITC-phalloidin were purchased from Sigma-Aldrich (St. Louis, MO). Horse serum was from Vector Laboratories (Burlingame, CA). Complete Freund's adjuvant (CFA) was obtained from Difco (Detroit, MI). Heparinase II was from Sigma-Aldrich (St. Louis, MO).

### Skin inflammation model

Skin Inflammation was induced in the flank of 8–12 week old female C57BL/6 recipient mice by three adjacent intradermal injections of CFA (2.5 mg/ml, 20 µl per injection). Mice were sacrificed 48 hours later and their inflamed skin tissue was removed and fixed. Naïve skin tissue was harvested from untreated mice.

### Immunofluorescence Procedures

Unless otherwise indicated, all skin sections were paraffin embedded. Skin specimens were fixed in 4% PFA and embedded in paraffin (4 µm sections). Target retrieval and deparaffinization were performed using the target retrieval solution, pH = 9 (Dako, Produktionsvej, Denmark) for 20 minutes at 95°C. Alternatively, skin specimens were fixed in 1% PFA followed by 30% sucrose and freezing. Cryostat skin sections (12 µm) were prepared and fixed in ice cold acetone for 10 min. All sections were similarly blocked with 10% horse serum, incubated with primary antibody at 4°C overnight (HS, VE-cadherin and LYVE1 1∶50, laminin 1∶100, α-SMA 1∶200, BS1–B4 1∶20), washed, and incubated with secondary antibody (1∶100) for 40 minutes at RT. Skin sections were imaged by a Delta-Vision microscope (Applied Precision, Issaquah, Washington). Inguinal lymph nodes were fixed overnight in buffer containing 0.05 M phosphate, 0.1 M l-lysine, pH 7.4, 2 mg/ml NaIO_4_, and 10 mg/ml PFA, and then dehydrated in consecutive sucrose gradients (10, 20, and 30% in phosphate buffer). Tissues were snap frozen in OCT compound Tissue-Tek® (Sakura Finetek). Frozen sections (6 µm) were stained with primary antibodies using the reagents and the protocol of the M.O.M kit (Vector Labs, Burlingame, CA). For some experiments, LN sections were pre-incubated for 60 min at 37°C with Heparinase II (1.5 SU/50 µl) diluted in 1% BSA/PBS, and then washed [14].

For analysis of HS deposition by skin derived endothelial cells (ECs) *in vitro*, Human Dermal Microvascular Blood Endothelial Cells (HDBEC) were cultured according to the supplier's instructions (Promocell Heidelberg, Germany), and plated at confluence on glass-bottom dishes spotted with fibronectin (20 µg/ml). ECs were stimulated with IL-1β (2 ng/ml) for 16–18 hours or left unstimulated. Samples were fixed with PBS containing 4% (wt/vol) paraformaldehyde and 2% (wt/vol) sucrose. For immunostaining of the basement membrane deposited by the ECs, fixed cells were permeabilized with saponin (0.1% (wt/vol)). Fixed cells were extensively washed, blocked with 10% (vol/vol) goat serum and incubated with primary antibody, followed by secondary antibody incubated with the nuclear dye, Hoechst, and the F-actin probe, phalloidin. Images were acquired using the Delta-Vision system (Applied Precision, Issaquah, WA). Sections were acquired as serial z stacks (0.2 µm apart) and were subjected to digital deconvolution (SoftWoRx, Applied Precision).

### Quantification of HS and BS1-B4 immunostaining

Fluorescence intensity was quantified with Image J (NIH). The ratio of fluorescence staining within apical and basolateral blood vessel compartments was extracted using a dedicated Matlab (Mathworks, Natick, MA) program. Based on the fluorescently labeled images, the algorithm quantified the red and green stained regions in the apical and basolateral sides of each separate endothelial cell, from which the area ratios for individual staining was deduced. To compare HS thickness among multiple resting and inflamed vessels, thickness values in the basolateral side of individual vessels were quantified across many locations along the vessel circumference using another dedicated Matlab program, and mean thickness values were calculated for each vessel.

### Statistical analysis

Reported values are expressed as mean with SEM (standard error of the mean). Statistical analysis was performed by Prism 5 software (GraphPad Software, Inc. La Jolla, CA) using Student's t-test. P value of <0.05 was considered significant.

## Results

### Resting skin vessels contain high HS densities at their basolateral compartments

**Figure 1:**
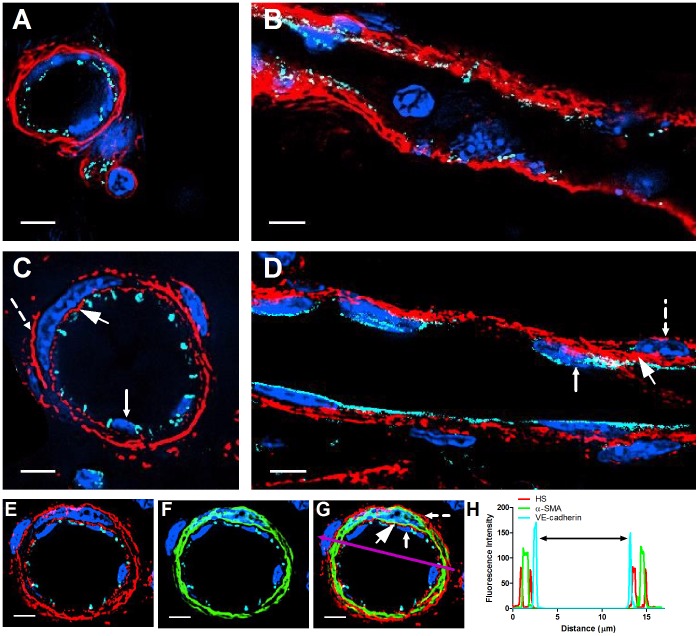
Resting skin vessels contain high density HS moieties preferentially expressed at their basolateral compartments. (A, C, E, F, G) Axial and longitudinal sections of vessels of (A, B) naïve frozen, or (C, D, E, F, G) paraffin embedded murine flank skin tissues. Tissues were stained for HS with monoclonal mouse IgM (10E4 epitope; red), goat anti mouse VE-cadherin (cyan), FITC-mouse monoclonal to α-SMA (green), and nuclei (blue). (H) HS and α-SMA profile of the fluorescence intensity along the purple arrow in the merged image. Double-headed black arrow denotes the vessel lumen as indicated by VE-cadherin. In C, D and G, small-headed arrows denote the endothelial apical side, large-headed arrows denote the endothelial basolateral side, and broken-line arrows denote the pericyte basolateral side. Images were taken at 100X magnification. Scale bar represents 5 µm.

We first took a semi-quantitative approach to assess *in vivo* the relative distribution of apical and subendothelial HS around resting venules within the flank skin. In this tissue, endothelial cells (ECs) can be readily depicted by VE-cadherin immunostaining, and post capillary venules were distinguished from other blood vessels by α-SMA immunostaining, since this protein is highly elevated on pericytes associated with post capillary venules (Figure S1). HS density and distribution were assessed with the pan HS specific IgM mAb 10E4, which recognizes N-sulfated glucosamine residues shared by all known HS GAGs [22]. Importantly, a recent study suggested that this N-sulfation rather than O-sulfation of the HS uronic acid moiety enables high affinity binding of chemokines [23]. Furthermore, this mAb does not bind hyaluronan, chondroitin sulfate (CS), dermatan sulfate, or keratan sulfate GAGs [22]. The specificity of this mAb was first confirmed *in vitro* by lack of 10E4 staining of CHO cells, which are deficient in xylosyl transferase and are unable to synthesize proteoglycans (Figure S2). We next analyzed 10E4 specific staining in both frozen and paraffin embedded sections from resting skin. Strikingly, all post capillary venules as well as arterioles exhibited much higher 10E4 reactivity at their basolateral compartments (basal lamina) compared to the endothelial apical side (lumenal) (Figure 1 and Figure S3). This HS high reactivity was detected both at the interface between ECs and their associated pericytes, as well as at the pericyte basolateral side (Figure 1E–H). This staining pattern suggested that HS GAGs are highly enriched in the basement membrane of skin post capillary venules, and raised the possibility that these vessels exhibit a sharp HS density gradient between their apical and sublumenal aspects.

### Heparan sulfate is diminished in the proximal BS1-B4 positive endothelial glycocalyx

**Figure 2:**
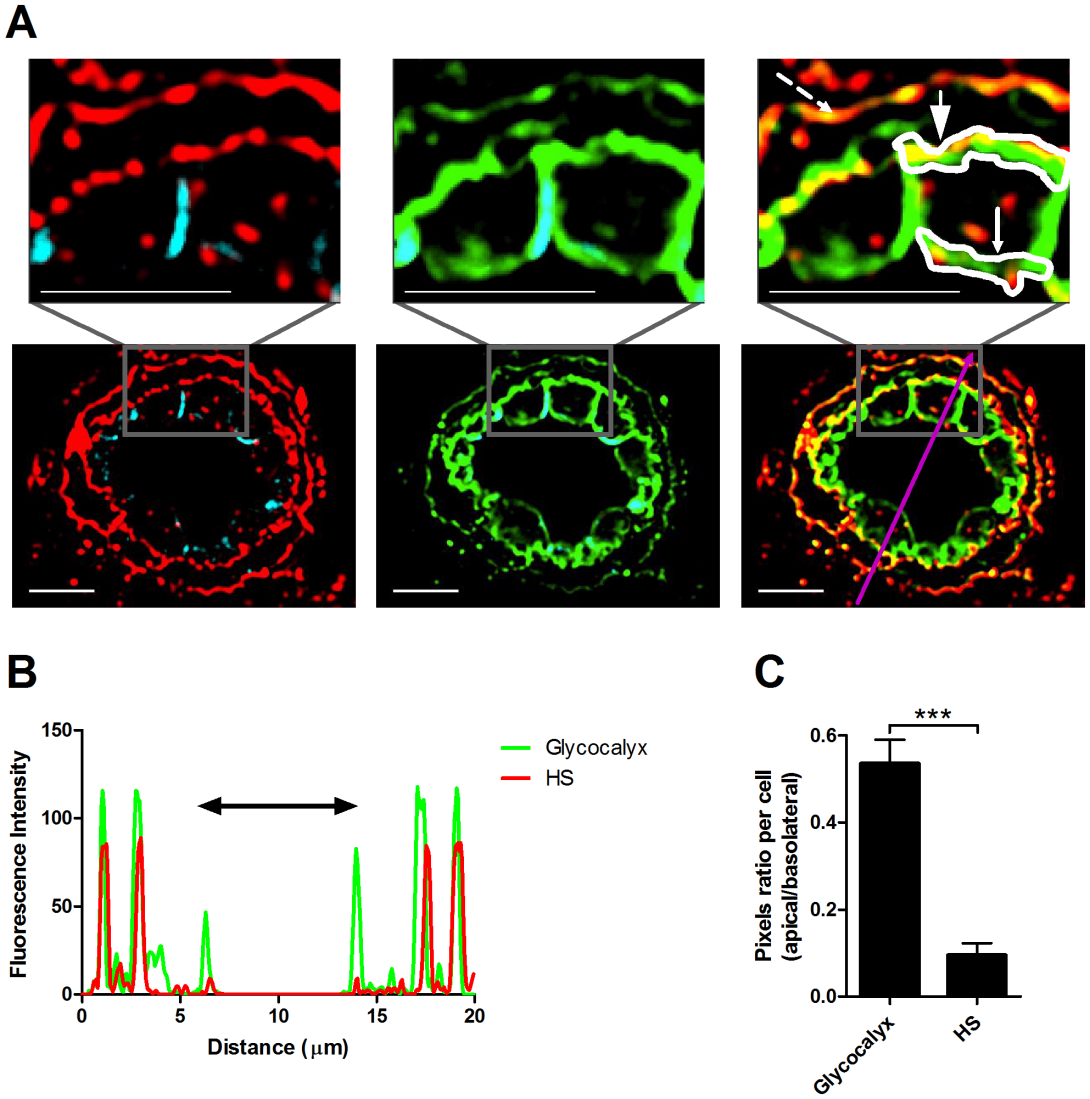
Low HS content within the apical glycocalyx of resting murine flank skin vessels. (**A**) Paraffin embedded murine naïve dermal tissue immunostained for HS (red; left panel), glycocalyx (α-gal) with the lectin BS1-B4 (green; middle panel), and VE-cadherin (cyan). Images were taken at 100X magnification. Scale bar represents 5 µm. (**B**) Glycocalyx and HS fluorescence intensity along the purple arrow in the merged image (right panel). Double-headed black arrow denotes the vessel lumen as indicated by the apical glycocalyx. (**C**) Pixel ratio per cell (apical/basolateral as indicated by the areas marked with white lines) in naïve murine dermal tissue. Small-headed arrow denotes the endothelial apical side, large-headed arrow denotes the endothelial basolateral side, and broken-line arrow denotes the pericyte basolateral side. Data are the mean value and S.E.M. of 16 endothelial cells analyzed in sections derived from three independent experiments. P<0.0001 by paired t-test.

Endothelial cells are decorated with variably sized glycocalyx layers composed of membrane bound glycoproteins and proteoglycans as well as non-covalently bound GAGs [24], [25], which in post capillary venules comprise an outer (distal) layer that can extend up to 0.5 µm away from the plasma membrane [26]. We first confirmed that the low HS content detected on the lumenal aspects of skin blood vessels was not the result of loss of covalently attached glycocalyx components from our samples. This was supported by considerable staining of the ubiquitous α-gal epitope detected by the BS1–B4 lectin (Figure 2A), a glycan probe used extensively for *in vivo* visualization of the lumenal glycocalyx of multiple types of blood vessels [27]. We next quantified the fluorescence intensity along the axial blood vessel (Figure 2B), and the pixel ratio of the apical to basolateral HS immunostaining, and compared it to the pixel ratio of apical and basolateral α-gal staining detected by the BS1-B4 lectin (Figure 2C). Whereas α-gal staining was almost uniformly distributed on the apical and basolateral compartments, HS staining was almost exclusively localized to the basolateral aspect of the resting skin vessels (Figure 2A–C). Collectively, our staining data strongly suggest that the proximal glycocalyx monolayer covering resting post capillary skin venules is largely devoid of HS, and that most of the HS associated with these blood vessels is confined to the basement membrane deposited by these blood vessels.

### The HS content of the basement membrane of skin post capillary venules is highly enriched under inflammatory conditions due to extensive deposition of ECM

**Figure 3:**
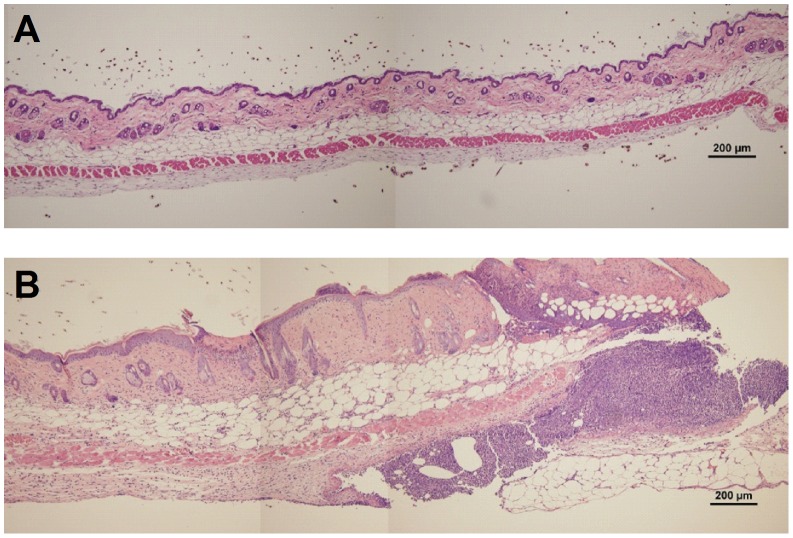
H&E staining of naïve and inflamed flank skin. (**A**) Naïve or (**B**) inflamed murine dermal tissue was stained and images were taken at 10X magnification. Scale bar represents 200 µm.

**Figure 4:**
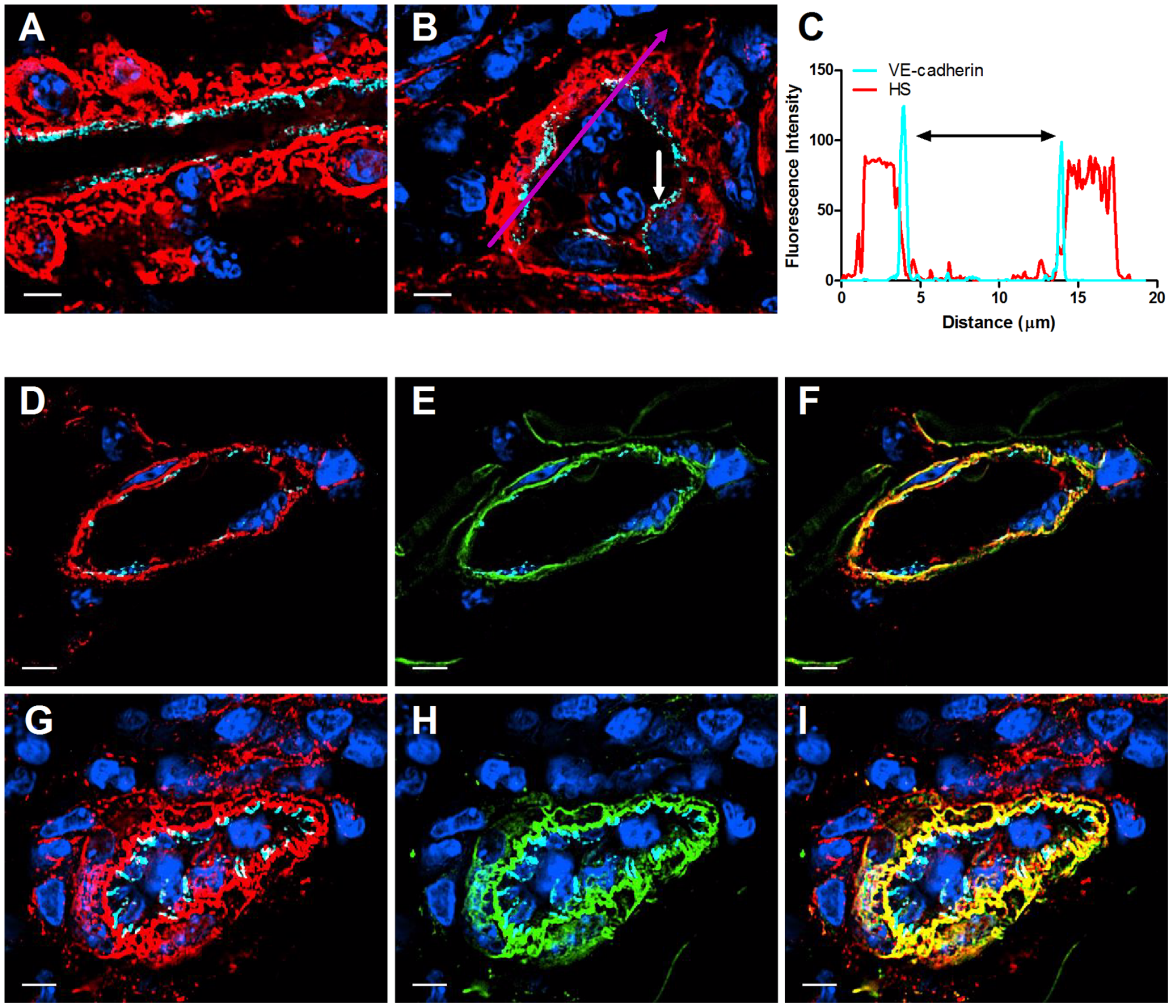
HS and laminin are massively deposited in the basement membrane of inflamed skin vessels. (**A**) Longitudinal and (**B**) axial paraffin sections of representative post capillary venules of CFA inflamed skin. (**C**) VE-cadherin and HS profile of the fluorescence intensity along the purple arrow in the axial section. Double-headed black arrow denotes the vessel lumen as indicated by VE-cadherin. In B, small-headed white arrow denotes the endothelial apical side. (**D, E, F**) Sections of naïve, and (**G, H, I**) inflamed murine dermal tissue, immunostained for HS (red), VE-cadherin (cyan), laminin with rabbit anti-mouse (green), and nucleus (blue). (**F, I**) merged images. Images were taken at 100X magnification. Scale bar represents 5 µm.

We next asked if inflamed skin vessels preserve the same HS content and polarized distribution found in resting venules. A progressive inflammation of the skin induced by local intradermal injection of complete Freund's adjuvant (CFA), resulted within 48 hours in swollen dermal tissue with substantial leukocyte infiltration (Figure 3). Notably, extensive basolateral deposition of HS was found around inflamed post capillary venules (Figure 4A–C), but not around arteries, venules or lymphatic vessels (data not shown). This massive HS enrichment in the basal lamina of inflamed post capillary venules was accompanied by co-enrichment of the basement membrane glycoprotein marker, laminin (Figure 4D–I). Interestingly, the basement membrane of resting or CFA inflamed skin post capillary venules did not contain fibronectin, and the global distribution of this adhesive glycoprotein within the inflamed interstitium remained comparable to that found in resting skin (data not shown), in contrast to reports on ear skin subjected to delayed type hypersensitivity reactions [28]. Since the ECM HS is expressed by proteoglycans rather than by glycoproteins [29], these results suggest that inflamed venules secrete extensive amounts of both HS-containing ECM proteoglycans as well as glycoproteins. The high co-localization of both HS moieties and laminin in disorganized networks also suggest that this extensive ECM deposition is orchestrated both by the inflamed ECs and their associated mural cells. Importantly, this inflammation-induced HS enrichment was not associated with a parallel increase in apical (lumenal) expression of HS moieties on inflamed skin vessels (Figure 4A–C). Thus, inflamed skin post capillary venules express much higher HS at their basolateral aspects than on their apical aspects under both resting and inflamed conditions.

### HS deposition near inflamed skin vessels is not elevated in mice deficient in the main HS degrading enzyme, heparanase

**Figure 5:**
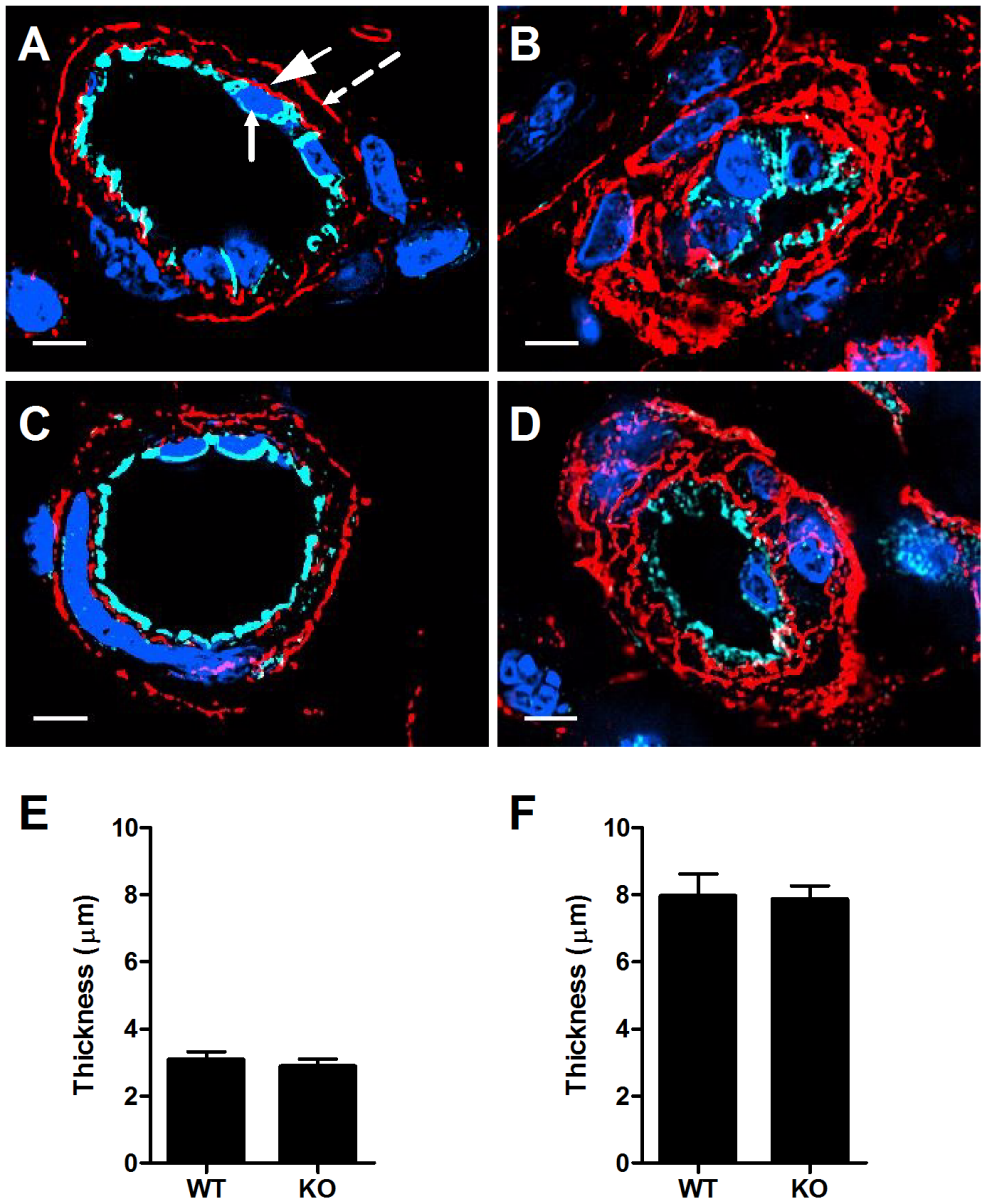
HS deposition is not elevated in mice deficient in the main HS degrading enzyme, heparanase. (**A, C**) Naïve, or (**B, D**) inflamed murine dermal tissue were removed from (**A, B**) wt or (**C, D**) heparanase KO mice, embedded in paraffin, and immunostained for HS (red), VE-cadherin (cyan), and nucleus (blue). Images were taken at 100X magnification. Scale bar represents 5 µm. In A the small-headed arrow denotes the endothelial apical side, the large-headed arrow denotes the endothelial basolateral side, and the broken-line arrow denotes the pericyte basolateral side. HS thickness (µm) in naïve (**E**), and inflamed (**F**) murine dermal vessels of either wt or heparanase KO mice. Data in each experimental group are mean value with SEM of 8 vessels in sections derived from three independent experiments.

Heparanase is a proinflammatory enzyme upregulated both transcriptionally and translationally by TNF or IL-1β signals in multiple types of cells including epithelial and ECs as well as in leukocytes [30]–[32]. We therefore reasoned that heparanase activities triggered within inflamed skin vessels and their surrounding stromal cells, including infiltrating leukocytes, may degrade the de novo deposited HS GAGs detected near inflamed skin venules. We also predicted that elevated HS would be detected near inflamed blood vessels of heparanase knock-out mice. Surprisingly, however, both inflamed and resting skin post capillary venules exhibited similar content and distribution of HS moieties (Figure 5A–F). Thus, heparanase activity in skin endothelium and in their closely associated stromal cells, as well as infiltrating leukocytes does not appear to modulate either the constitutively expressed or the de novo deposited HS GAGs in skin tissues.

### Human dermal microvascular blood endothelial cells deposit HS mainly at their basolateral aspect in a cell autonomous manner

**Figure 6:**
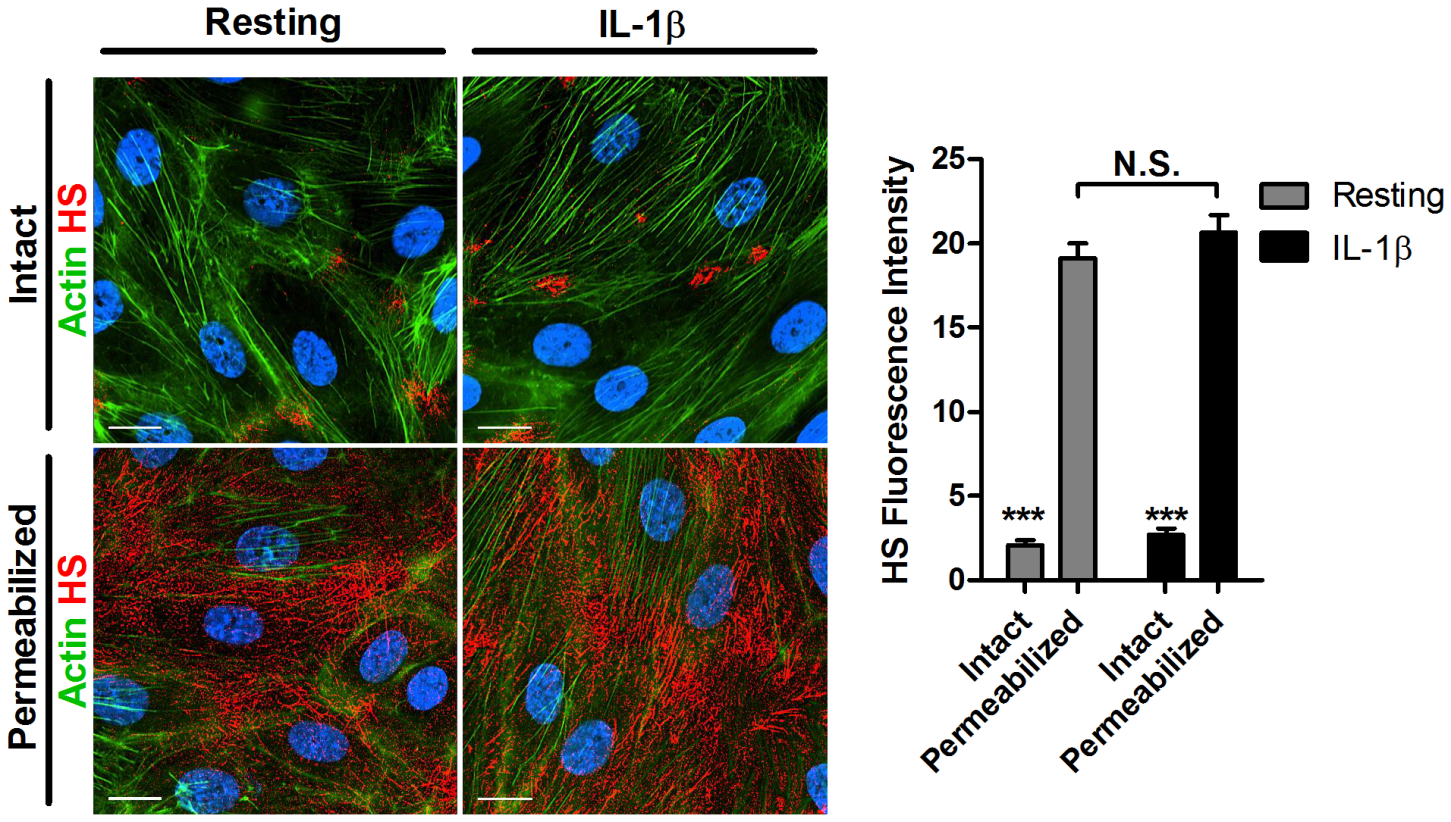
HS is localized mainly to the basement membrane in both resting and inflamed human dermal microvascular blood endothelial cells (HDBECs). Immunostaining for HS (red), actin (green) and nuclei (blue) of resting or IL1-β- stimulated HDBEC monolayers grown for 16–18 hours on fibronectin. The mean fluorescence intensity values shown are the mean±SEM of ten fields of view from three independent experiments. Images were taken with a Delta-Vision microscope at 60X magnification. Scale bar represents 15 µm. N.S. not significant. ***P<0.0001 by Student's t test.

The highly enriched HS detected at the basement membranes of skin post capillary venules could arise from both endothelial deposited HS, as well as pericyte secreted HS (Figure 1E–H). In addition, the capacity of ECs to pattern HS gradients across their apical and basolateral aspects could depend on paracrine signals from pericytes and other perivascular cells. We therefore took an *in vitro* approach to test whether the HS enrichment in the basement membranes of skin blood vessels reflects a cell-autonomous capacity of isolated cultured dermal ECs to deposit high HS preferentially at their basement membranes. To that end, we analyzed the HS distribution on either intact or permeabilized confluent HDBEC monolayers with the 10E4 anti-HS IgM mAb. Without permeabilization the accessibility of this large mAb to the basolateral aspects of the endothelial monolayer was negligible, such that the vast majority of the mAb specific staining was constrained to the apical endothelial surface (Figure 6). After endothelial permeabilization the IgM could readily access the basolateral compartments of the endothelial monolayers. Using this differential staining approach we detected much higher HS staining within the basolateral aspects than on the apical aspects of the HDBEC monolayers (Figure 6). Notably, the HS pattern, distribution and level of expression detected by the 10E4 mAb were not altered after IL-1β stimulation (Figure 6). Thus, the dramatically elevated levels of HS deposited *in vivo* within the basement membrane of inflamed skin post capillary venules (Figure 4), likely reflect a combined contribution of HS secretion by inflamed ECs, inflamed pericytes, as well as other perivascular resident cells. Taken together these *in vitro* results suggest that the capacity of ECs to pattern HS gradients between their apical and basolateral aspects is cell autonomous. Furthermore, the *in vitro* and *in vivo* results collectively suggest that the enhanced deposition of HS rich ECM “carpets” during inflammation *in vivo* (Figure 4) around skin vessels is contributed both by endothelial and non-endothelial perivascular counterparts.

### HS is highly enriched in the basal lamina of lymph node HEVs

**Figure 7:**
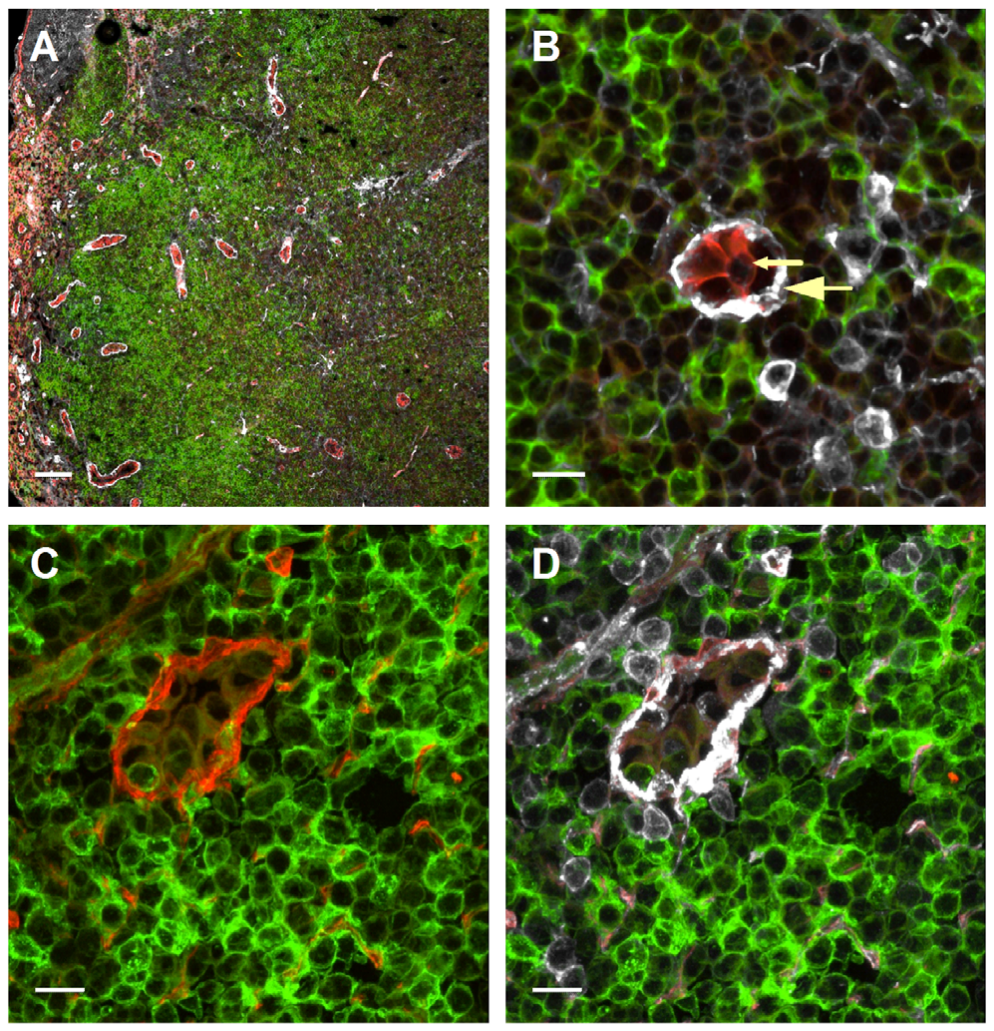
HS is highly enriched in the basal lamina of lymph node HEVs. (**A, B**) Cryosections of naïve lymph nodes derived from ROSA^mT/mG^ × CD11c-Cre reporter mice immunostained for HS (grey). CD11c+ cells express the GFP protein (green), and endothelial cells express high levels of the tomato protein reporter (red). (**C, D**) Immunostaining of similar lymph node sections for HS (grey) and laminin (red). In B, small-headed arrow denotes the endothelial apical side as indicated by the tomato staining, and large-headed arrow denotes the endothelial basolateral side. Images were taken at (**A**) 10X magnification (scale bar represents 100 µm), and (**B, C, D**) 63X magnification; scale bars represent 10 µm.

**Figure 8:**
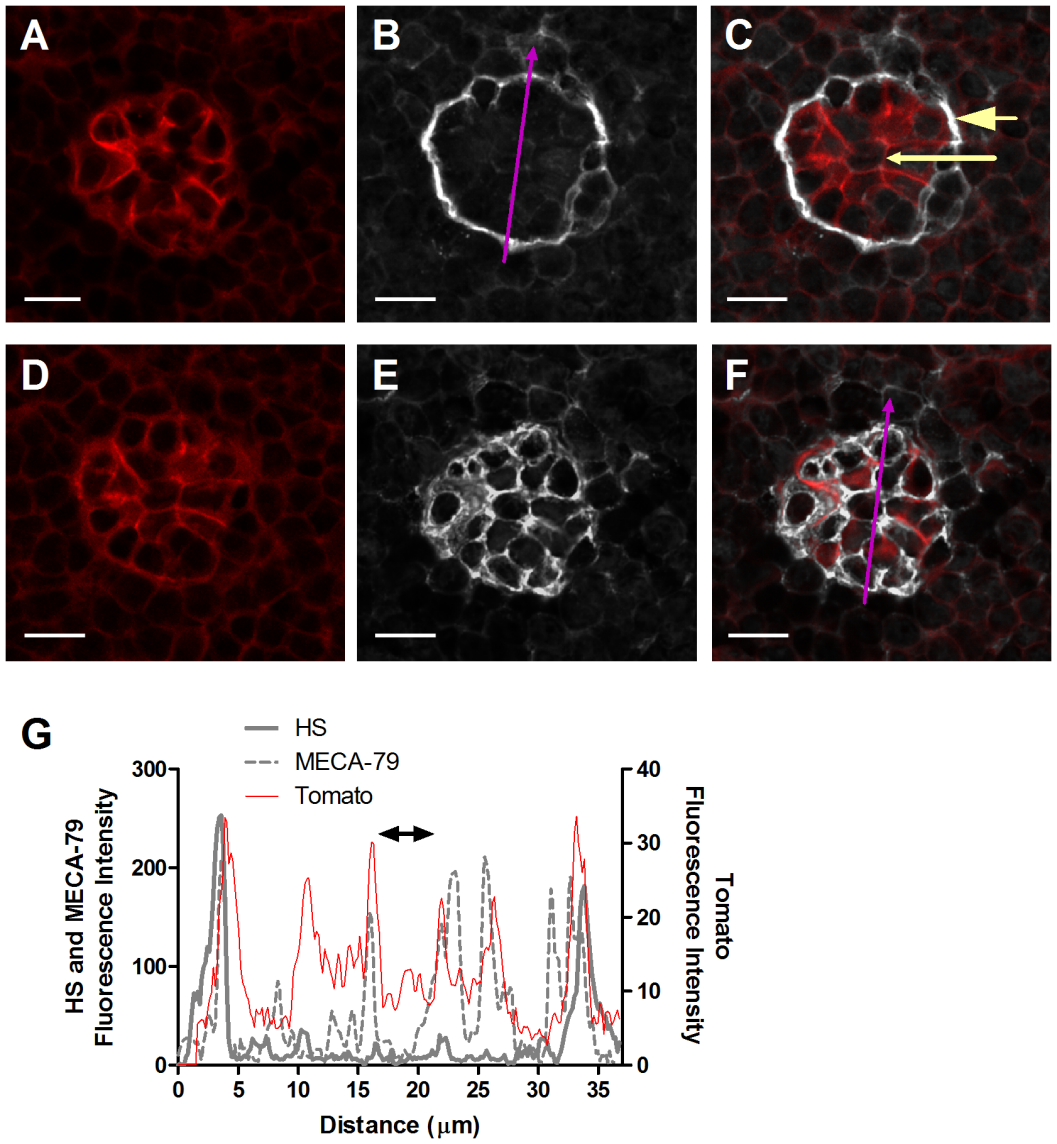
Differential distribution of HS and of PNAds in lymph node HEVs. Consecutive sections of lymph node cryosections of the ROSA^mT/mG^ × CD11c-Cre reporter mice stained for (**B, C**) HS (grey), or (**E, F**) PNAd (grey) using the 10E4 or MECA-79 IgMs, respectively. (**A, C, D, F**) Note the intense tomato protein label on the HEVs (red). (**C, F**) Merged images. (**G**) HS and MECA-79 profile of the fluorescence intensity along the purple arrow. Double-headed black arrow denotes the vessel lumen as indicated by the tomato staining. In C, small-headed arrow denotes the endothelial apical side, and large-headed arrow denotes the endothelial basolateral side. Images were taken at 63X magnification. Scale bars represent 10 µm.

Lymph node HEVs are the primary post capillary venules that support massive constitutive lymphocyte recruitment [33]. The CCR7 chemokines CCL21 and CCL19 have been implicated in lymphocyte recruitment and diapedesis across these vessels, and surface-bound CCL21 has been reported to be highly enriched at the basolateral aspects of HEVs [11], [13]. The high levels of CCL21 and of other lymphocyte attracting chemokines, including CXCL13 and CXCL12 within the basal lamina of HEVs were attributed to their affinity for collagen IV [34]. Recent studies suggested, however, a key involvement of HS biosynthesis by HEVs in both T cell arrest and entry to resting lymph nodes, consistent with a critical role of HS-dependent chemokine presentation by these ECs in lymphocyte crossing of HEVs [13]. We therefore hypothesized that similar to skin post capillary venules, a steep gradient of HS might exist between the apical and basolateral aspects of lymph node HEVs. We therefore took a similar HS staining approach to that used in the skin, but rather than VE-cadherin staining, we used ROSA^mT/mG^ CD11c-Cre transgenic mice expressing ‘tomato’ and GFP markers to distinguish HEVs from lymph node DCs. The ROSA^mT/mG^ strain was crossed with the CD11c-Cre transgenic mouse strain [21] resulting in membrane-targeted green fluorescent protein (mG) expression in all CD11c cells. The tomato reporter was ubiquitously high on all blood vessels and could be easily distinguished from the GFP labeled CD11c+ cells allowing visualization of these two major cell types in fixed samples at single cell resolution. As shown in Figure 7A–D, all HEVs of naive inguinal lymph nodes appeared bright red, in contrast to the abundant DCs surrounding these HEVs which appeared green. Both membrane-targeted markers also highlighted membrane structures, and permitted visualization of fine cellular processes (Figure 7B). Thus, immunostaining of these sections with the HS specific mAb 10E4 allowed us to determine at high spatial resolution the density of HS moieties found on either the apical and sublumenal interface of the bright red ECs. Specificity of the mAb staining was confirmed by total loss of 10E4 staining after Heparinase II digestion (Figure S4). As observed in the skin, almost all the HS detectable around HEVs was localized to the basal lamina deposited by these cells, with essentially undetectable HS staining on the lumenal aspects (Figure 7A, B). In agreement with their enrichment in the basal lamina, HS staining colocalized with laminin (Figure 7C, D). Interestingly, no BS1-B4 staining could be detected on either the apical or the basolateral aspects of HEVs (data not shown), suggesting major differences in the biochemical composition of the glycocalyx surrounding the skin versus the lymph node post capillary venules. We therefore used MECA-79 as a specific marker of the HEV glycocalyx. As expected, MECA-79 stain was noted on all HEVs and this glycocalyx layer was clearly distinguished from the endothelial cell body and enriched at the lumenal, junctional and basolateral aspects of individual HEVs (Figure 8D–F). Nevertheless, no HS staining could be found on the lumenal side of HEVs (Figure 8A–C), as could also be seen by the quantification of the fluorescence intensity along the axial blood vessel (Figure 8G), further substantiating the restricted expression of HS in the HEV basement membrane. These results collectively suggest that although biochemically distinct in their glycan composition, both non lymphoid and lymphoid post capillary venules share a similar pattern of highly enriched HS GAGs concentrated at the basolateral (ablumenal) aspects of these vessels.

## Discussion

Cytokine stimulated post capillary venules are the major blood vessels through which leukocytes traffic into tissues [4]. Multiple adhesive ligands cooperate with chemokines presented on both the apical and basolateral aspects to coordinate leukocyte adhesion to, and motility on and across the endothelial barriers that comprise these vessels. It is believed that net chemotactic gradients between the apical and basolateral aspects of the post capillary venules are essential for the highly directional nature of leukocyte diapedesis across the vessel wall, but the evidence for such net gradients is still weak.

Despite the growing evidence that immobilized rather than soluble chemokines are the functional chemoattractive unit critical for directional leukocyte migration in the interstitium [14], it has been unclear to date whether and how soluble and surface immobilized chemokines produced by ECs and their adjacent stromal cells generate and maintain stable gradients across blood vessels i.e., from the apical to the basolateral aspects of blood vessel barriers, thereby promoting leukocyte diapedesis [35]. Notably, surface bound chemokine gradients can form in interstitial spaces containing uniform HS scaffolds [17], and thus, the generation of such gradients seems to depend on local chemokine production as well as on rapid degradation, deactivation, or scavenging of excess chemokines [36]. In contrast, endothelial produced or transported chemokines can be readily washed from the lumenal aspects of blood vessels by blood flow, and therefore, pure mass action may be sufficient to sustaining sharp gradients between apical and basolateral endothelial surfaces. Here, we provide the first evidence of an additional regulatory mechanism to generate and sustain stable chemokine gradients, employed by multiple types of ECs; this mechanism involves robust HS enrichment within the basement membranes of both resting and inflamed post capillary venules.

Our present study puts in perspective several previous findings that indicated strong HS GAG staining around ECs [13], [15], [37]. Due to low resolution, the relative distribution of HS GAGs around the blood vessels studied in these reports appeared to be uniformly expressed on both flat ECs and in lymph node HEVs. The improved resolution attained here, allowed us to differentiate between the endothelial cell body and its immediate glycocalyx and ECM in these two prototypic post capillary venules, and led us to conclude that endothelial HS is mainly concentrated within the basal lamina the endothelial monolayer. Thus, in addition to their well-known cytokine storage activity, mechanical stabilizing functions [38], as well as restricting endothelial permeability to transport of solutes and proteins [39], basement membrane HS proteoglycans deposited by ECs may be critical for the proper positioning of HS-binding chemokines to leukocyte protrusions which scan endothelial junctions for exit cues [40], [41]. Basement membrane HS GAGs may also protect chemokines secreted by endothelial, perivascular stromal cells, as well as recently transmigrating leukocytes from proteolytic degradation [42].

Endothelial glycocalyx composition and structure within intact, inflamed and enzyme-treated blood vessels [43] was previously compared by multiple approaches including *in vivo* dextran exclusion measurements, lectin staining, fluorescence microparticle image velocimetry [44], as well as lanthanum-based electron microscopy [45]. These studies suggest that this highly hydrated gel-like structure is composed of two main layers: a compact inner layer <100 nm above the apical membrane surface, and an outer layer ranging up to 0.5 µm away from the plasma membrane [26]. This outer layer contains extended core proteins, mechanically stabilized by HS and CS GAGs [24], [29], [43], [46], and continuously secretes HA chains that are non-covalently tethered to the apical endothelial CD44 [24]. Multiple inflammatory signals have been traditionally suggested to induce the shedding of this endothelial glycocalyx layer, and such shedding may be necessary for the exposure of transmembranal adhesion molecules ordinarily masked by endothelial glycocalyx [27], [31], [43], [47]–[49]. The membrane proximal glycocalyx layer, on the other hand, is composed of both non GAG glycoproteins as well as membrane-bound GAG proteoglycans such as HS and CS, containing syndecans and glypicans, which connect to the endothelial cortical cytoskeleton or to apical endothelial caveolae, respectively [24]. Due to its small dimensions, this thinner layer is probably the exclusive layer through which rolling leukocytes can encounter and establish adhesive interactions with their endothelial expressed integrin ligands.

In our hands, considerable BS1–B4 staining was readily detectable within a layer 0.3–0.5 µM away from the endothelial cell body, demonstrating that the fixation protocols used by us retained considerable fraction of the endothelial glycocalyx. This notion allows us to conclude that the apical glycocalyx layer of skin post capillary venules contains much lower HS content than the HS moieties deposited within the basolateral endothelial compartment of these vessels, both under resting and inflamed conditions.

In spite of their low density and large distance from the plasma membrane, specialized apical endothelial HS moieties, must be able to stably present, alone or with another scaffold molecule such as DARC or a related molecule [50], [51], sufficient HS binding chemokine molecules to rolling leukocytes so they can activate their integrins and arrest on the target endothelial surface. The low availability of apical HS GAG scaffolds for chemokines identified by us in the present work raises the possibility that once arrested on the apical endothelial membrane, some leukocytes may need to integrate post arrest signals from endothelial produced or transcytosed chemokines in an HS independent manner, i.e. via confined release of intra-endothelial chemokine pools within submicron synapses generated by the endothelial adherent leukocytes and the endothelial surface they serially engage [52]. These pools of chemokines may be particularly efficient in their ability to locally activate leukocyte GPCRs, and trigger leukocyte protrusions at the apical endothelial surface. A subset of these protrusions would then transverse the endothelial junctions and respond to basolateral chemokines presented by the dense HS moieties of the endothelial basement membrane.

Although inflammatory conditions were suggested to increase HS degradation by its main degrading enzyme, heparanase, our studies in the inflamed skin suggest that inflammation in fact dramatically elevates HS and laminin deposition by post capillary venules. Furthermore, although heparanase levels and activities are upregulated in many inflammatory settings [53]–[55], and *in vivo* siRNA silencing of heparanase induces heparanase dependent changes in skin vessel permeability [53], this massive deposition of HS containing proteoglycans near inflamed vessels is not mitigated by heparanase activity. Thus, both at steady state and after induction of local skin inflammation, both heparanase knock-out mice and wt mice shared similar HS enrichment near skin blood vessels suggesting that this enzyme is not functional within resting or inflamed skin vessels.

In conclusion, our results support the notion that both peripheral and lymph node post capillary venules preferentially express HS-containing scaffolds within their respective basement membranes, rather than in the thin plasma membrane glycocalyx decorating the immediate adhesive layer formed by their transmembranal glycoproteins. Importantly, this pattern of highly polarized HS expression seems to be conserved in multiple types of post capillary venules. Together with the extensive washing of chemokines presented on the lumenal endothelial aspects, this polarized HS expression may be responsible for the generation of chemokine gradients between the lumenal and basolateral aspects of blood vessel barriers that participate in leukocyte recruitment to target tissues. During inflammation, post capillary venules in the skin and possibly in other inflamed tissues can further enrich the HS content of their perivascular compartments, generating additional HS scaffolds in the form of HS rich ECM “carpets”. This highly polarized production of HS GAGS may result in two major physiological outcomes: it could help blood vessels sustain a net gradient of HS scaffolds for chemokines that bind surface GAGs, and thereby sustain steep chemokine gradients for leukocytes emigrating across inflamed vessels; in addition, this dense perivascular HS patterned by post capillary venules may also present key pro-inflammatory cytokines to recently emigrated leukocytes. These HS GAGs may contribute to the capacity of such cytokines to reprogram the navigation and effector machineries of emigrating leukocytes [56] as they pass through the basal lamina. Further dissection of their composition and functions may therefore identify novel targets for anti-inflammatory therapy.

## Supporting Information

Figure S1 **α-SMA staining is observed around post capillary venules and arterioles, but not near lymphatic vessels.** Immunofluorescence of (**A**) post capillary venule, (**B**) arteriole, and (**C**) lymphatic vessel of naïve murine skin. (**A, B, C**) Paraffin sections stained with FITC-mouse monoclonal to α-SMA (green), goat anti-mouse VE-cadherin (cyan), nuclei (blue), (**C**) rabbit anti mouse LYVE1 (blue). Images were taken at 100X magnification. Scale bar represents 5 µm.
(TIF)

Figure S2 **10E4 staining of WT and proteoglycan-deficient CHO cells.** (**A**) WT and (**B**) HS deficient CHO cells stained for HS (red) and nuclei (blue). Images were taken at 20X magnification. Scale bar represents 40 µm.
(TIF)

Figure S3 **Arterioles deposit HS at their basolateral compartment.** An arteriole in paraffin section of naïve murine skin, stained for α-SMA (green), HS (red), VE-cadherin (cyan) and nuclei (blue). (**C**) Merged image. Images were taken at 100X magnification. Scale bar represents 5 µm.
(TIF)

Figure S4 **Elimination of HS staining in lymph node HEVs after Heparinase II digestion.** Lymph node cryosections of the ROSA^mT/mG^ x CD11c-Cre reporter mice stained for HS (grey) (**A**) without, or (**B**) after Heparinase II treatment. CD11c+ cells express the GFP protein (green). Endothelial cells express high levels of tomato protein reporter (red). Images were taken at 25X magnification. Scale bars represent 50 µm.
(TIF)

Data S1 **Cell culture conditions of Chinese hamster ovary (CHO) cells.**
(DOCX)
