# Supplementary figures and images for: Blood Vessels Pattern Heparan Sulfate Gradients between Their Apical and Basolateral Aspects
Source: PLoS One. 2014 Jan 22;9(1):e85699. doi: 10.1371/journal.pone.0085699 (PMC3899079; doi:10.1371/journal.pone.0085699)

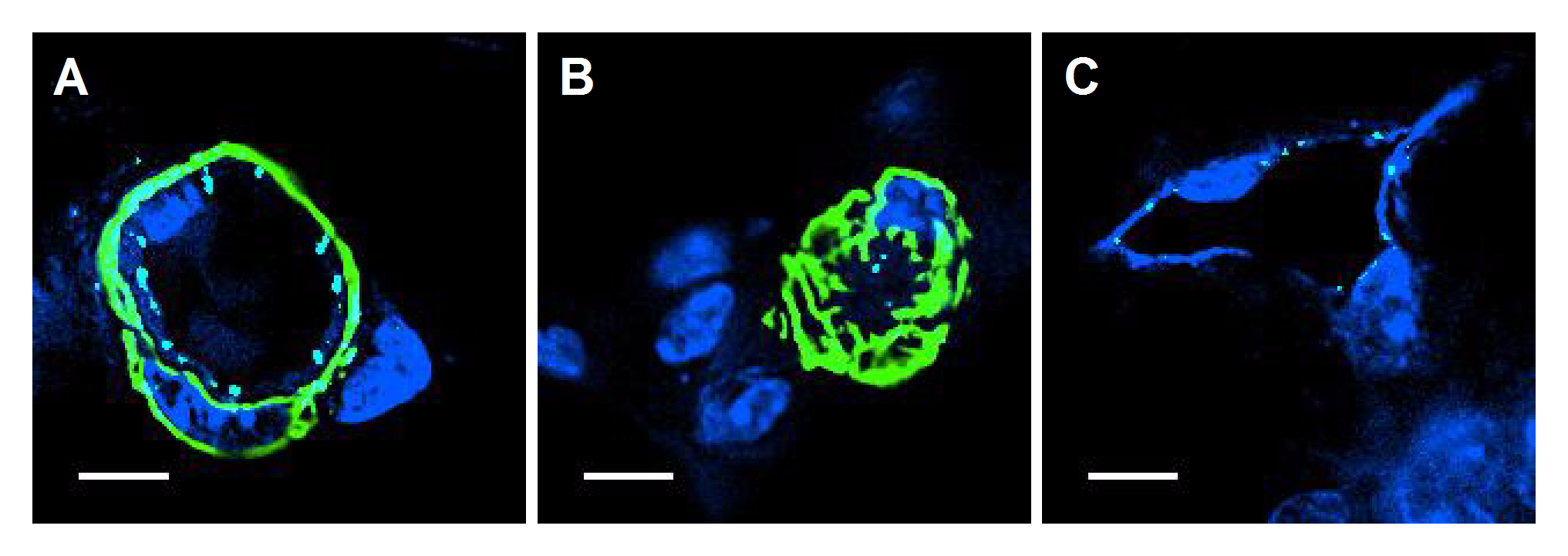

Supplement: Figure S1 — α-SMA staining is observed around post capillary venules and arterioles, but not near lymphatic vessels. Immunofluorescence of (A) post capillary venule, (B) arteriole, and (C) lymphatic vessel of naïve murine skin. (A, B, C) Paraffin sections stained with FITC-mouse monoclonal to α-SMA (green), goat anti-mouse VE-cadherin (cyan), nuclei (blue), (C) rabbit anti mouse LYVE1 (blue). Images were taken at 100X magnification. Scale bar represents 5 µm. (TIF) [file pone.0085699.s001.tif]

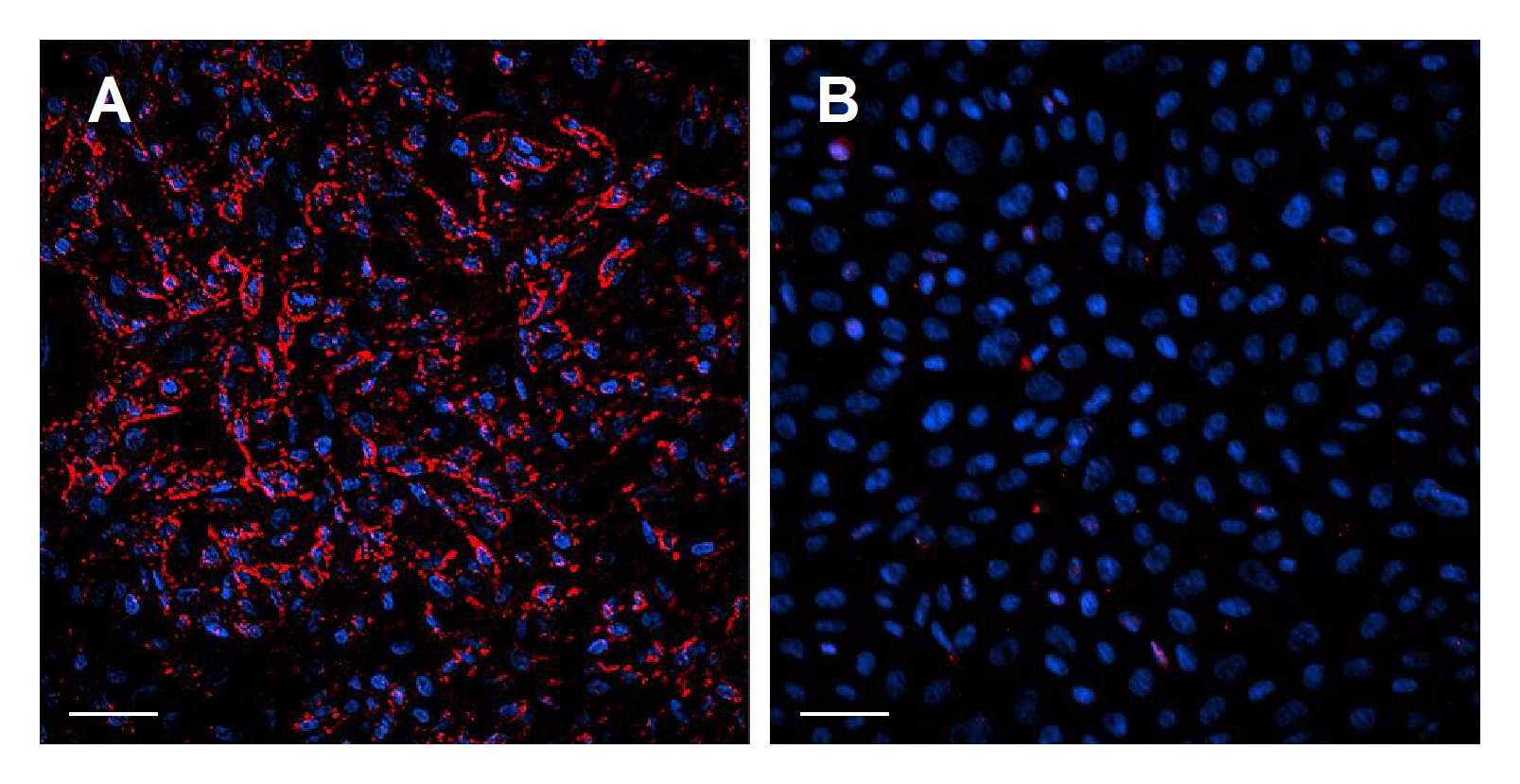

Supplement: Figure S2 — 10E4 staining of WT and proteoglycan-deficient CHO cells. (A) WT and (B) HS deficient CHO cells stained for HS (red) and nuclei (blue). Images were taken at 20X magnification. Scale bar represents 40 µm. (TIF) [file pone.0085699.s002.tif]

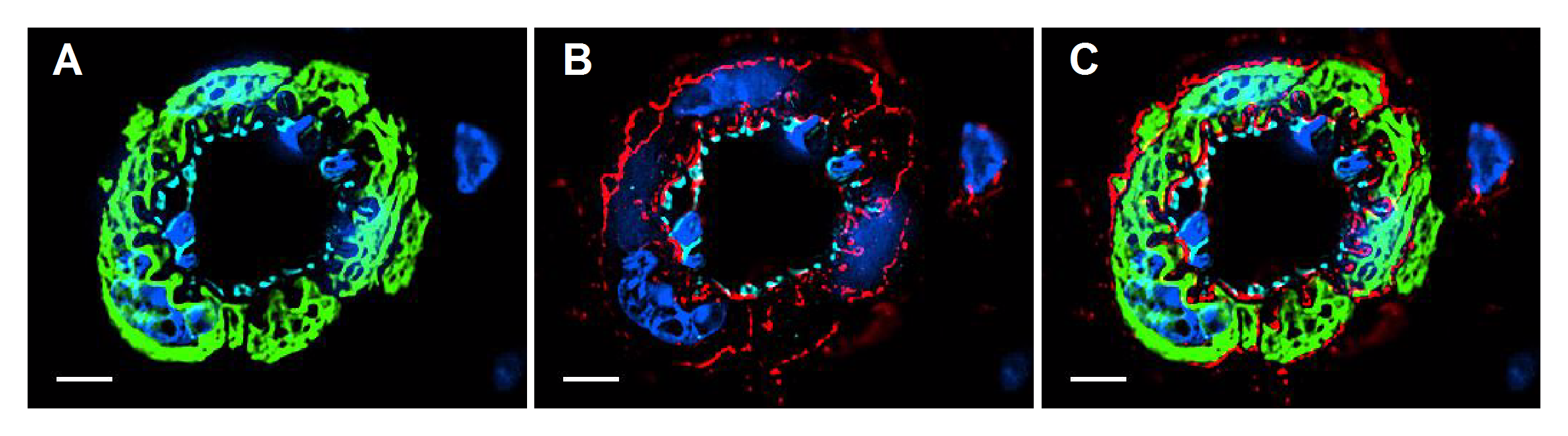

Supplement: Figure S3 — Arterioles deposit HS at their basolateral compartment. An arteriole in paraffin section of naïve murine skin, stained for α-SMA (green), HS (red), VE-cadherin (cyan) and nuclei (blue). (C) Merged image. Images were taken at 100X magnification. Scale bar represents 5 µm. (TIF) [file pone.0085699.s003.tif]

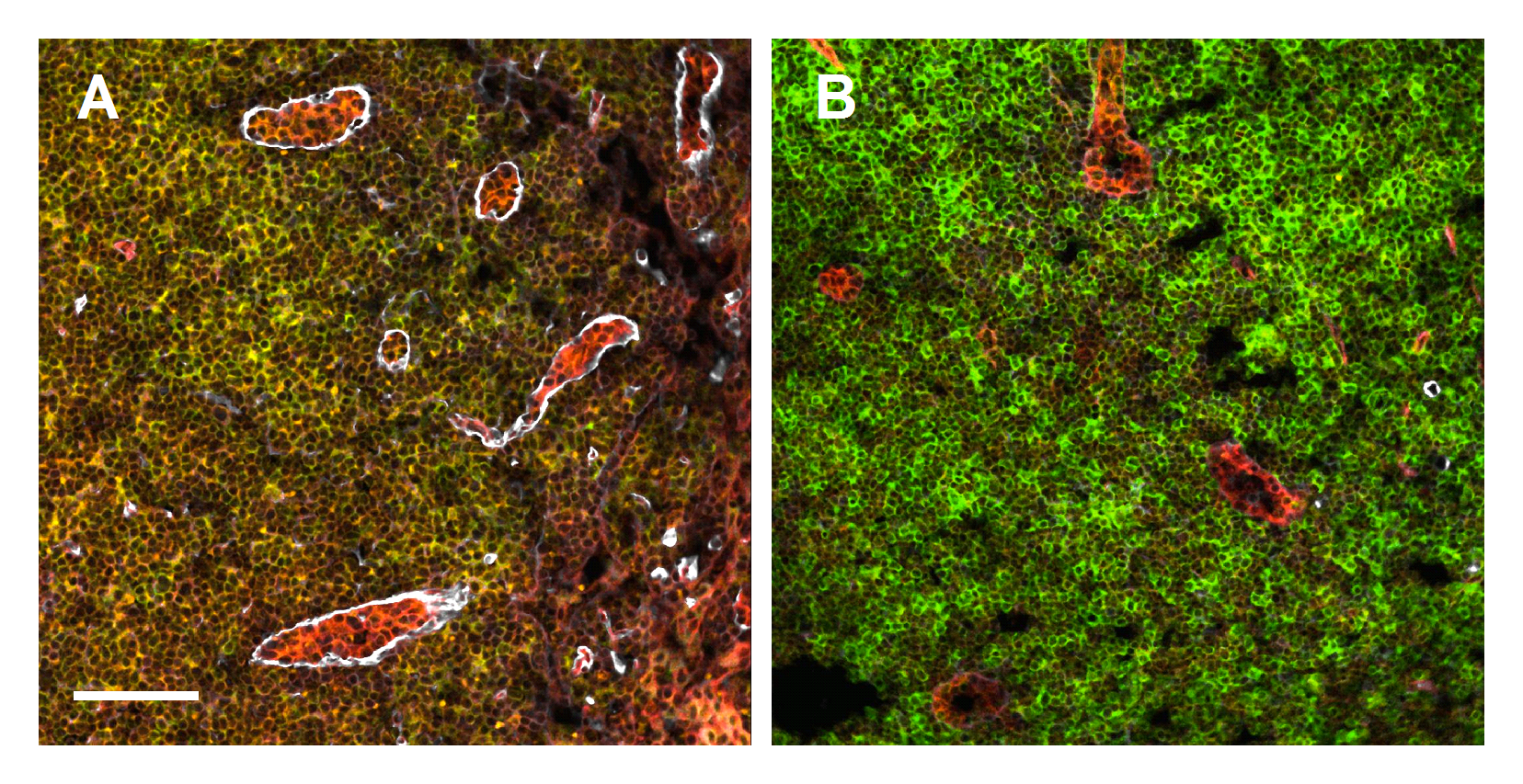

Supplement: Figure S4 — Elimination of HS staining in lymph node HEVs after Heparinase II digestion. Lymph node cryosections of the ROSAmT/mG x CD11c-Cre reporter mice stained for HS (grey) (A) without, or (B) after Heparinase II treatment. CD11c+ cells express the GFP protein (green). Endothelial cells express high levels of tomato protein reporter (red). Images were taken at 25X magnification. Scale bars represent 50 µm. (TIF) [file pone.0085699.s004.tif]
