# Supplementary material for: Blood Vessels Pattern Heparan Sulfate Gradients between Their Apical and Basolateral Aspects
Source: PLoS One. 2014 Jan 22;9(1):e85699. doi: 10.1371/journal.pone.0085699 (PMC3899079; doi:10.1371/journal.pone.0085699)
Supplement: Data S1 — Cell culture conditions of Chinese hamster ovary (CHO) cells. (DOCX) [file pone.0085699.s005.docx]

**Materials and methods**

**Cells**

Wild-type Chinese hamster ovary (CHO) cells CHO-k1 and xylosyl transferase deficient CHO-pgA-745 (mutant) cells were kindly provided by Prof. I. Vlodavsky (Technion, Haifa). Cells were grown in DMEM (wild-type) or in RPMI (mutant) containing 2mM L-glutamine, 1mM sodium pyruvate, 10% FCS, and antibiotics, all purchased from Biological Industries (Beit Haemek, Israel).
